# The Gut Microbiota: Master of Puppets Connecting the Epidemiology of Infectious, Autoimmune, and Metabolic Disease

**DOI:** 10.3389/fmicb.2022.902106

**Published:** 2022-04-29

**Authors:** Olaf F. A. Larsen, Maike van der Grint, Cato Wiegers, Linda H. M. van de Burgwal

**Affiliations:** Athena Institute for Research on Innovation and Communication in Health and Life Sciences, Vrije Universiteit Amsterdam, Amsterdam, Netherlands

**Keywords:** gut microbiota, epidemiology, infectious disease, autoimmune disease, metabolic syndrome, antibiotics usage, vaccination

## Abstract

Infectious, autoimmune, and metabolic diseases put an enormous pressure on both quality of life and the economy. For all three disease types, it is known that the quality of the gut microbiota composition is correlated to both onset and progression of disease. Hence, maintaining eubiosis and preventing gradual irreversible loss of beneficial microbes within the gut microbial ecosystem is of utmost importance. As such, the epidemiological trends of these disease types may serve as proxies for the integrity of the human gut microbiota. Here, we present incidence data covering the last decades for prototypical infectious diseases (tuberculosis and measles), autoimmune disorders (type-1 diabetes and multiple sclerosis), and the prevalence of metabolic syndrome. Our findings reveal that vaccination efforts correlate with relatively low levels of archetypal infectious disease incidence. However, autoimmune and metabolic disorders are, together with the usage of antibiotics, steeply on the rise. These findings suggest that the status of the gut microbiota is persistently deteriorating, as reflected by the proxies. As such, the epidemiological trends shown here may serve as a starting point for a mechanistic understanding of the interplay between these different disease types that can be used for future prevention and mitigation strategies like targeted stimulation and suppletion of microorganisms by means of, e.g., fermented foods, prebiotics and probiotics.

## Introduction

The twenty-first century boldly demonstrates that infectious diseases are far from eradicated, exemplified by emerging zoonotic diseases like SARS, MERS and, most notably, COVID-19 (Allen et al., 2017; Hu et al., 2021). The impact of this trend cannot be underestimated. Currently, the SARS-CoV-2 pandemic has resulted in about 6 million deaths globally (WHO, 2022), while the global population growth is expected to decelerate, caused by, among other factors, continued flare ups of COVID-19 (World Bank, 2022). On top of that, it is predicted that more infectious diseases will emerge, driven by processes like international travel and trade, labor migration and increasing population density (Jones et al., 2008; Elias et al., 2021). Hence, new strategies that efficiently tackle these emerging pandemics, like the proposed blockchain-facilitated sharing of research efforts (van der Waal et al., 2020), are of utmost importance to warrant quality of life and economic stability for the future.

While infectious diseases are generally spread by the transmission of pathogenic microorganisms between humans, zoonotic diseases like COVID-19 involve not only interhuman interactions but also other ecosystems like soil and animals. As such, it is a prerequisite to get a thorough understanding of both the pathogenic and mutualistic microbial interactions crossing these different domains in order to effectively prevent and manage possible new outbreaks (Larsen and van de Burgwal, 2021). For instance, it is nowadays well established that a balanced gut microbiota (eubiotic state) is a prerequisite for developing an adequate immune response system (Maynard et al., 2012). Proper maintenance of the gut microbiota requires continuous replenishment of microorganisms, and hence one should prevent diminished exchange of beneficial microorganisms between different domains. Especially in early childhood, limited exposure to specific microorganisms (“old friends”) is indicated to give rise to suboptimal functioning of the immune system (Rook and Brunet, 2005). Indeed, earlier results showed that a diminished exposure to microorganisms in early life is correlated to a higher susceptibility to health problems like atopic diseases and behavioral problems (Von Mutius et al., 2000; Dawson et al., 2021).

The integrity of the human gut microbiota is shown to be correlated with the susceptibility to and the outcome of infectious, metabolic, and autoimmune diseases (Libertucci and Young, 2019). This has become even more pronounced with the onset of the SARS-CoV-2 pandemic. Right at the start of the pandemic, the gut microbiota was suggested to influence the course of COVID-19 disease (Dhar and Mohanty, 2020). Recently, a cohort study indeed demonstrated that the gut microbiota composition is linked with disease severity for patients with COVID-19 (Yeoh et al., 2021). Moreover, in line with metabolic diseases being (causally) related to the gut microbiota (Fan and Pedersen, 2021), SARS-CoV-2 infection is now being recognized as exacerbator of metabolic disease complications (le Roux, 2021). Vice versa, while dysbiosis of the gut microbiota has been linked to autoimmune disease development before (Wu and Wu, 2012; Xu et al., 2019), it is now shown to also increase the risk for developing COVID-19 (Akiyama et al., 2021).

Taken all this, the recent historic trends in infectious, autoimmune, and metabolic disease should be investigated, as these data can serve as proxies for our exposure to beneficial microbes. This exposure contributes to the integrity of the gut microbiota and hence our resilience toward disease in general. Insights into disease trends are urgently needed; earlier perspectives already warned for the consequences of a gradual loss of our gut microbes (Blaser and Falkow, 2009). Population-wide losses of beneficial microbes are not only dependent on improved hygiene, but also on the usage of antibiotics. The consequences of which are not limited to reduced resilience against (newly emerging) infectious diseases, as Bach’s seminal 2002 paper already showed a rapid increase in the incidence of autoimmune diseases like Type-1 Diabetes (T1D) and asthma in the Western world during the last 50 years of the twentieth century. This increase was accompanied by a profound decline in the incidence of “classical” infectious diseases like tuberculosis and measles, attributed to improved hygiene and vaccination programs (Bach, 2002).

Therefore, we inventoried the incidence of various archetypical, “classical” infectious diseases for the last 40 years, providing an update of the data presented in Bach (2002). With “classical” we exclude newly emerging infectious disease like SARS and COVID-19, as their historical data only covers very few years, hence having limited statistical validity. Next to this, we collected incidence data of several autoimmune diseases during the same time period, as well as the prevalence of metabolic disease. These data were put in perspective by data on antibiotics usage and vaccination coverage, which we also analyzed.

## Materials and Methods

### Study Demarcation and Data Collection

Similar to Bach (2002), we included data from a number of countries in Western Europe and the United States. The countries from Western Europe comprised Finland, France, Germany, Italy, the Netherlands, and the United Kingdom, to cover the north-south gradient within Western Europe that may attribute to differences in autoimmune disease incidence (Bach, 2002). Data from 1980 to 2020 were covered in this study, to be able to make a comparison with trends presented by Bach (2002) regarding the twentieth century, as well as provide insight into whether and, if so, how these trends continue into the twenty-first century.

The epidemiological data of two infectious diseases, two autoimmune diseases and one metabolic disease were included in this study. Tuberculosis (TB) was chosen to represent infectious diseases as the disease is still prevalent in most countries and the pathogenesis is similar to COVID-19. Data on TB was extracted from the 2020 Global Tuberculosis report of the World Health Organization (WHO, 2020a), which contained the number of reported cases for all included countries from 1980 until 2019. Also, data on vaccine coverage was included to provide a clear overview of the effects of vaccination. The Bacillus Calmette–Guérin (BCG) vaccine against TB is currently not mandatory in any of the investigated countries, however, in Germany, Finland, and France, this was the case until 1998, 2006, and 2007, respectively (Zwerling et al., 2011).

Data on measles was also collected, as this infectious disease can be considered archetypical in its mode of transmission as well as in being vaccine preventable. Presently, measles vaccination is highly recommended in Finland, the Netherlands, and the United Kingdom and mandatory in Germany, France, Italy and the United States (Our World in Data, 2021). Therefore, analogous to the data on BCG vaccine coverage, data on Measles-Containing-Vaccine dose 1 (MCV1) coverage was included as well. The WHO vaccine-preventable diseases monitoring system (WHO, 2020b) was used to retrieve data on measles incidence, and BCG and MCV1 vaccination coverage since 1980 for the included countries. The data were further supplemented with data from Vanderslott et al. (2019), providing information on vaccine coverage in years not included in the WHO data.

T1D and Multiple Sclerosis (MS) were included as archetypical autoimmune diseases due to their different characteristics. Whereas T1D is an autoimmune disorder that is mostly diagnosed at a young age, MS is a neurological disorder that is usually diagnosed in a later stage of life and progresses during the course of life (Milo and Miller, 2014). For T1D, several published articles provided data on the incidence in the included countries or representative regions thereof during varying time periods [United States: 2001–2015 (Rogers et al., 2017), Netherlands: 1999 and 2011 (Fazeli Farsani et al., 2016), Finland: 1984–2005 (Harjutsalo et al., 2008), 2006–2011 (Harjutsalo et al., 2013), 2014 and 2018 (Parviainen et al., 2020), France: 2010–2015 (Piffaretti et al., 2019), Germany and United Kingdom: 1993–2013 (Patterson et al., 2019), Italy: 1992–2003 (Bruno et al., 2010)]. When the incidences of T1D in representative regions were provided, a weighted average was calculated to estimate the incidence rate for the entire country. Most studies focused on children between the age of 0–14 years, however, two studies (Fazeli Farsani et al., 2016; Rogers et al., 2017) included all children below the age of 19. As most cases of T1D are diagnosed in children around 13–14 years old (CDC, 2021), we did not consider this to significantly skew the data. For MS, individuals who were newly diagnosed at all ages were included. The Atlas of MS created by the MS International Federation (MSIF) provided data on the incidence of MS at three points in time (2008, 2013, 2020) for the countries included in this study (MSIF, 2020).

The prevalence and incidence of metabolic diseases are growing at an alarming rate, and metabolic syndrome (MetS) in particular has been deemed an epidemic (Vrdoljak et al., 2022). MetS data were included to gain insight into the growing problem of and to compare trends in the incidences of infectious, autoimmune, and also metabolic disease. Data on MetS prevalence in the United States were derived from articles based on the Centers for Disease Control and Prevention (CDC) National Health and Nutrition Examination Survey (NHANES) and consisted of data on individuals ≥20 years of age, suffering from three or more indications required for the diagnosis of MetS (Aguilar et al., 2015; Marcotte-Chenard et al., 2019; Liang et al., 2021). As the NHANES was performed biannually since 1999, only data from 1999 to 2018 were included in this study.

Antibiotics are frequently used as a highly effective treatment for bacterial infections, but they can also lead to antimicrobial resistance (AMR) or infections with opportunistic pathogens (Raplee et al., 2021). Data on antibiotics usage were included to study its effect on the incidence of infectious, autoimmune and metabolic disease from 1980 to 2020 as well as provide insight into possible developing trends of antimicrobial resistance. Data on the use of antibiotics was retrieved from the antimicrobial consumption database (ESAC-Net) of the European Centre for Disease Prevention and Control (ECDC, 2020). This database uses the Anatomical Therapeutic Chemical (ATC) classification system. Anti-infectives classified as ATC J, consisting of all antibacterials, antimycotics, antimycobacterials, and antivirals for systemic use, were included in this study. To the best of our knowledge, no data could be found for the United States and for the period 1980–1996. Additionally, since data on antibiotics use in the hospital sector were largely incomplete, this study only included antibiotics use specified by the ECDC as “community (primary care sector).” For clarity, in this study the term antibiotics refers to antimicrobials in general and not solely to antibacterials.

### Data Analysis

In this study the incidence was determined, as it provides crucial information on the rate at which the disease is spreading in case of infectious diseases (Williams and Wright, 1998). The incidence of autoimmune diseases can indicate if the disease is becoming a more significant problem for public health. If the incidence of autoimmune diseases remains stable, this indicates that there is no change in factors that influence the development of the disease. However, if the incidence of autoimmune diseases is increasing, this means that either factors are changing that influence the development of the disease, or detection methods have improved. For both MS and T1D, there are no new methods to diagnose disease that have been widely incorporated in the period of 1980–2020.

Based on the available newly reported cases of each disease for each country every year, the incidence per 100,000 inhabitants in that year was calculated. Data on the annual population of each of the included countries were retrieved from the World Population Prospects by the United Nations (2019). Since the data on T1D is based on the incidence of the disease in 0–14-year-olds, the incidence was not measured per 100,000 inhabitants, but rather per 100,000 person-years. This unit of measurement indicates the total amount of time contributed by each individual while they remained at risk (Boskey, 2020). For MetS, the prevalence is displayed, rather than the incidence. This is because individuals with MetS are not diagnosed at one identifiable point since it is an accumulation of symptoms. Additionally, as one of the included studies on MetS prevalence presented data per ethnicity (Marcotte-Chenard et al., 2019), a weighted average was calculated based on the ethnicity data of the United States population included in that study. The vaccination coverage for BCG against TB and MCV1 against measles is displayed as the percentage of 1-year-olds that were immunized that year. Regarding antibiotics use, according to the ECDC, the antibiotics consumption is measured in Defined Daily Dose (DDD) per 1,000 inhabitants per day.

To enhance clarity of the data visualization, trendlines were presented. For data on the incidence of T1D and MS, as well as the percentage of the United States population with MetS and antibiotics consumption in European countries, a linear trendline was added as a guide for the eye. For the incidence data on TB and measles, an exponential trendline was added as a guide for the eye. These trendlines were constructed by calculating the average value of all included countries per year and formulating a linear or exponential equation based on the values of all available years. Significance of a trend (based on averages) was calculated using the Mann Kendall Trend Test performed in R (R Core Team, 2020).

## Results

### Incidences of Prototypical Infectious Diseases Decrease to Low Plateau Values

**FIGURE 1:**
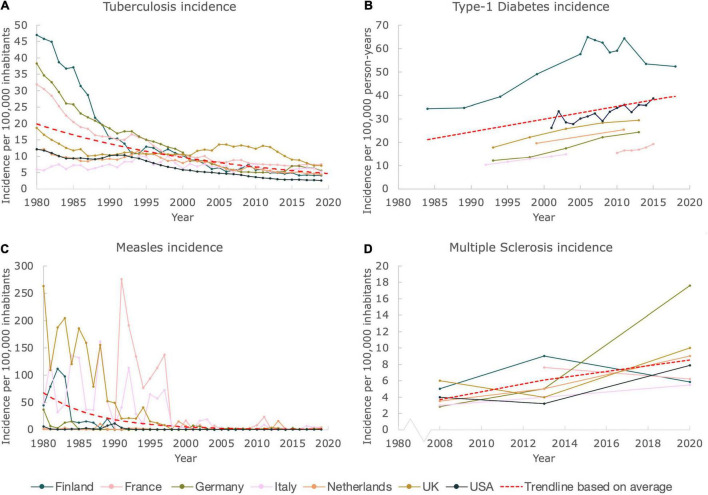
While incidences of prototypical infectious diseases declined, incidences of autoimmune diseases increased. Figure shows incidences of prototypical infectious and autoimmune diseases for Finland, France, Germany, Italy, the Netherlands, United Kingdom and United States. The trendlines (exponential for TB and measles incidences, and linear for T1D and MS incidences) are based on the average values, and merely serve as a guide to the eye. A Mann Kendall Trend Test was performed for all figures (except for the data on MS due to the limited amount of data). The test showed significance: *P* < 0.05, for all incidences (except for the MS data, which did not allow a test to be performed). **(A)** The incidence of TB from 1980 until 2019. **(B)** The incidence of T1D from 1984 until 2018. **(C)** The incidence of measles from 1980 until 2019. **(D)** The incidence of MS from 2008 until 2020.

The incidences (measured in numbers of new cases per 100,000 inhabitants) of two common infectious diseases, TB and measles were constructed for several Western European countries as well as for the United States, for the time period 1980–2019. As displayed in Figures 1A,C, the incidences of both diseases in selected countries rapidly decreased during this time period. For TB, the incidence in Italy remained relatively stable, being ∼6 in 1980, and ∼5 in 2019. Contrastingly, the incidences in Finland, Germany and France declined drastically during the last four decades, with Finland having an incidence of ∼47 in 1980, that lowered steeply to ∼4 in 2019. For the Netherlands, the United Kingdom and the United States, this lowering in incidence was also observed, albeit less pronounced since initial 1980 values were already low (19, 12, and 12 respectively). The data of the last 5 years of analysis did not show any particular trend up- or downwards, suggesting a plateau is reached, with incidences lower than 10 for all countries.

As shown in Figure 1C, measles has, just like TB, almost been eradicated in the selected countries. The incidence data show a pronounced decrease for all countries. Flare ups of the virus are clearly visible during the timespan 1980–2000. When zooming in on the last two decades, only minor temporary outbreaks of the virus are detected. Nevertheless, in comparison to measles incidences in the twentieth century, the associated numbers are very minor, with the highest incidence being ∼24 in 2011 in France, as compared to ∼276 in 1991, indicating a tenfold decrease.

### Incidences of Prototypical Autoimmune Diseases Anticorrelate With the Incidences of Common Infectious Diseases

Data on the incidences of autoimmune diseases was more challenging to retrieve, as compared to the data of infectious diseases. Several articles on the incidences of T1D of children between 0–14 and 0–19 years old (for the Netherlands and United States only) were found and included in this study. Although data was not available for every year for the countries studied, a clear upward trend was observed when merging the available data, as can be seen in Figure 1B. The incidence in Finland seems to be declining after steadily rising until ∼2005. Nevertheless, also for Finland an increase in the incidence was clearly observed when examining the total timespan from 1980 to 2019. We included a linear trendline based on the average values for clarity.

The Atlas of MS provided a clear and nearly complete dataset on the incidences of MS for the total population of the studied countries for the years 2008, 2013, and 2020. Just like for T1D, a convincing trend upward can be observed, which is also confirmed by the linear trendline that we included (Figure 1D), based on the average values. Using this trendline it can be estimated that the MS incidence on average doubled between 2008 and 2020, for the countries selected. In 2008, the average incidence equaled ∼4, whereas a number of ∼9 was found in 2020.

### The Prevalence of MetS in the United States Persistently Rises to Almost 40%

**FIGURE 2:**
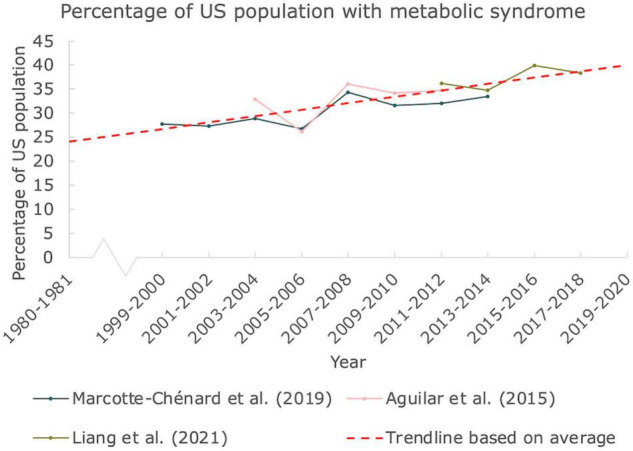
Prevalence of MetS in the United States. The percentage of people in the United States suffering from at least 3 indications responsible for MetS. Data derived from 3 articles based on NHANES by CDC. A linear trendline was added as guide to the eye (Mann Kendall Trend test: *P* < 0.05).

In Figure 2, the prevalence of MetS was constructed from three articles published in scientific literature, all three based on the CDC NHANES. All articles used provided the percentages of individuals ≥20 years old with three or more indications for MetS. A trendline was constructed based on average values for clarity as well. The percentage of the United States population suffering from MetS has been increasing over the past two decades. While the prevalence in the year 2000 was already close to 30%, it reached close to 40% in 2018, emphasizing the immense scale of this problem.

### Antibiotics Usage Shows Moderate Increase Over the Last Decades in Europe

**FIGURE 3:**
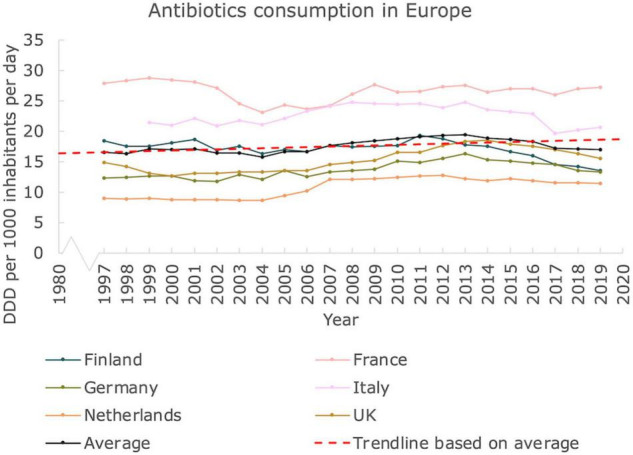
Antibiotics usage in Europe. Antibiotics consumption in Defined Daily Dose per 1000 inhabitants and per day in Western European countries, from 1997 until 2019. A linear trendline was added as guide to the eye (Mann Kendall Trend test: *P* < 0.05).

To get insight on antibiotics usage and possible associated AMR, we provide an overview of antibiotics consumption in Western European countries for the period 1997–2019 (Figure 3). The antibiotics consumption, as defined by the DDD per 1,000 inhabitants and per day seems relatively stable. According to the trendline we constructed, based on the average, a moderate increase of ∼0.09 DDD per year is seen. Hence, based on this number, an increase from ∼16.5 DDD on average in 1997 to ∼18.5 DDD in 2019 on average can be deduced, corresponding to an increase of ∼12%. Interestingly, the countries with the highest DDD are located in Southern Europe (Italy, France) whereas the more Western and Northern European countries displayed a lower DDD. Data on antibiotics consumption from the United States could not be retrieved.

### Vaccination Coverage Is Associated With the Stabilization of Infectious Disease Incidence

**FIGURE 4:**
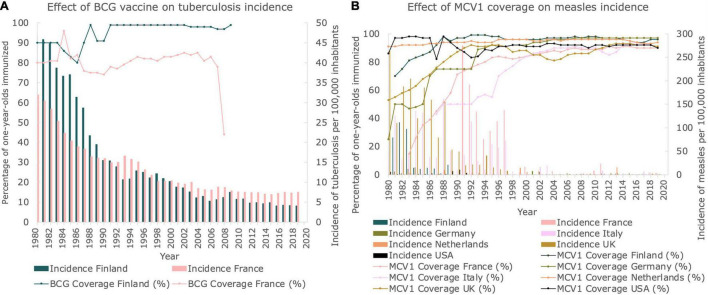
Vaccination coverages for TB and measles. **(A)** Vaccination coverage of BCG vaccine and corresponding incidence of TB in Finland and France between 1980 and 2019. Line graphs represent the BCG vaccination coverage. Bar graphs represent the incidence of TB per 100,000 inhabitants. **(B)** Percentage of 1-year-olds immunized with MCV1 per country between 1980 and 2019. Line graphs represent the MCV1 vaccination coverage. Bar graphs represent the incidence of measles per 100,000 inhabitants.

We investigated vaccination coverages for both TB and measles in the countries studied. Finland, France and Germany included the BCG vaccine in their vaccination program, inducing immunity against TB. As can be seen in Figure 4A, the vaccination coverage of Finland was close to 100% (in fact 98%) for 1-year-olds immunized from 1991 until 2006, when the mandatory vaccination stopped. In France, the vaccination coverage was lower, fluctuating between 77 and 85% during the same time period until mandatory vaccination stopped in 2007. When these countries stopped vaccinating with BCG, the number of new cases stabilized at approximately 4 and 7 new cases per 100,000 inhabitants per year, respectively. Although the BCG vaccine was also mandatory in Germany until 1998, no vaccination coverage data could be retrieved.

In Figure 4B, the percentage of immunized 1-year-olds from each country studied is presented for the measles-containing-vaccine dose-one (MCV1). In 1980, vaccination coverage of MCV1 was relatively high for both the Netherlands and the United States (91 and 86%, respectively), whereas vaccination coverages as low as 25% were observed for Germany. However, in 2014, at least 90% of all 1-year-olds in all the countries studied was vaccinated, and a stabilization of vaccination coverage was observed. When comparing the incidences of measles with the MCV1 vaccine coverage, both displayed in Figure 4B, a correlation between reaching a plateau in incidence and reaching a plateau in vaccination coverage can be seen, which approximately sets in at the year 2000.

## Discussion

Our data demonstrate that prototypical autoimmune disease incidence is still steadily increasing and accompanied by a plateauing decline in prototypical infectious disease incidence. Moreover, our data show a strong association between the level of eradication of infectious disease and the population coverage of vaccination programs. Two decades after Bach’s seminal paper indicating a clear link between reduced infectious disease incidence and increased autoimmune and allergic disease incidence (Bach, 2002), the trend has not been reverted but rather continues into the twenty-first century. These results are a reinforcement of the epidemiological trends observed in the twentieth century, that were interpreted as resulting not only from effective vaccination programs and improved hygienic standards, but also from diminished exchange of beneficial microorganisms between different domains. Decreased exposure especially in early childhood, is recognized to give rise to a disproportional immune response towards these beneficial microbes (“old friends”) in later life, contributing to the onset of autoimmune type indications (Rook and Brunet, 2005).

Our data demonstrate a sharp increase in autoimmune disease, as exemplified by the incidences of T1D and MS. In general, autoimmunity is considered to be a predisposition for infectious disease (Maddur et al., 2010). The autoimmune indication T1D increases the severity of COVID-19 symptoms significantly, just like its metabolic counterpart Type-2 Diabetes (T2D) (Gregory et al., 2021). However, recent meta-analyses suggest that, in general, autoimmune diseases do not lead to a more severe outcome of COVID-19 (Liu et al., 2020; Druyan et al., 2021). The comorbidities that patients with autoimmune indications may suffer from, as well as the immune therapies these patients receive, can, however, lead to increased vulnerability to COVID-19, as was recently shown for MS patients (Chaudhry et al., 2021; Reder et al., 2021).

Next, our data show a profound increase in prevalence of MetS in the United States, being close to 40% in 2018. Obesity and metabolic syndrome are known to impact immunity, leading to increased susceptibility to infectious disease, its associated disease progression and vaccine efficacy (Karlsson and Beck, 2010; Andersen et al., 2016). Moreover, perturbations in the cross talk between the immune and metabolic system may lead to autoimmune disease (Zmora et al., 2017; Medina et al., 2018). Recent data obtained by CDC has shown that for hospitalized COVID-19 patients, the prevalence of metabolic indications ranked highest (up to ∼50%) when listing underlying comorbidities, with essential hypertension, disorders in lipid metabolism, obesity, diabetes with complication, and atherosclerosis and other heart disease comprising the top 5 of indications (Gundlapalli, 2021). SARS-CoV-2 is now being recognized as a facilitator of metabolic disease complications.

Antibiotics usage can lead to the propagation of AMR genes in the human microbiota—the resistome—and the societal effects are staggering. A recent study showed that over 1.2 million deaths in 2019 can be attributed to bacterial AMR (Murray et al., 2022). As the resistome is inherited maternally as well as developed throughout life (Pärnänen et al., 2018), this problem is likely to further exacerbate in the coming years. While the rise of AMR is an alarming health problem in and of itself, the complications of antibiotic-induced changes to the gut microbiota are more profound. Associations between antibiotic usage and conditions as diverse as obesity, diabetes (T1D and T2D), and asthma have been described (see Patangia et al., 2022 for an overview). Considering that the gut microbiota is strongly correlated to metabolic indications (Fan and Pedersen, 2021) and also to autoimmune disorders (Xu et al., 2019), it is suggested that a gradual decrease of the quality of the gut microbiota during the last decades is the common factor in explaining both metabolic and autoimmune incidence data. Importantly, however, our data show that despite relatively higher use of antibiotics in France and Italy, these countries report lower incidence rates of autoimmune diseases such as MS and T1D. This reinforces the notion that the effect of antibiotics on the incidence of autoimmune diseases is multifactorial and should be considered in comprehension (Bach, 2021).

While improved hygiene measures have contributed to a reduction in prototypical infectious diseases, simultaneously, a sharp increase in the number of emerging and re-emerging infectious diseases is seen across the globe. A progressively deviating microbiota composition has been indicated in disturbed immune maturation, leading to reduced response to pathogenic infections (Patangia et al., 2022). In light of this, the association between both disturbed microbiota composition and reduction of known pathogenic infections with the rise in autoimmune diseases, should be further investigated (Bach, 2021). Promising results of functional foods in the management of the intestinal resistome should also be viewed in this light (Tsigalou et al., 2020). It remains to be seen whether these changes to the gut microbiome enable hosts to maintain sufficient resilience against infections with newly emerging infectious diseases. Moreover, considering the wide implementation of COVID-19 hygiene measures it is of interest to study the effect on the incidence of autoimmune and infectious diseases in the years to follow. Early declines in influenza infections might be a canary in the coalmine, indicating reductions in the spread of beneficial as well as pathogenic microbes as a result of social distancing measures (Sullivan et al., 2020).

The primary strength of this study is that, to the best of our knowledge, it shows for the first time the most complete overview of the available epidemiological data on MS, TB, T1D, MS, and MetS for several Western European countries and the United States, from 1980 till 2020. A limitation is that there is no direct evidence yet whether and to what extent these epidemiological trends are being explained by a diminished exposure to beneficial microbes. For example, the data on the incidence of TB and measles will be a convolution of both vaccination efficacy as well as other factors like a diminished microbial exposure due to excessive hygiene.

To conclude, our information serves as an update of the data already provided in 2002 by Bach, providing us with a more complete picture also covering the last decades. As such, these data can provide a first step toward further unraveling a mechanistic understanding of the interplay between these different disease types in the context of different (microbial) ecosystems. This serves as a starting point for the development of future prevention and mitigation strategies.

## Data Availability Statement

Publicly available datasets were analyzed in this study. This data can be found here: United Nations (2019), Vanderslott et al. (2019), ECDC (2020), MSIF (2020), and (WHO, 2020a,b).

## Author Contributions

OL: conceptualization, writing, and editing. MG: analysis and writing. CW and LB: reviewing and editing. All authors contributed to the article and approved the submitted version.

## Conflict of Interest

The authors declare that the research was conducted in the absence of any commercial or financial relationships that could be construed as a potential conflict of interest.

## Publisher’s Note

All claims expressed in this article are solely those of the authors and do not necessarily represent those of their affiliated organizations, or those of the publisher, the editors and the reviewers. Any product that may be evaluated in this article, or claim that may be made by its manufacturer, is not guaranteed or endorsed by the publisher.
